# Minimal residual disease testing in gastrointestinal cancers: a US perspective

**DOI:** 10.1093/gastro/goag111

**Published:** 2026-11-06

**Authors:** Ryne C Ramaker, John H Strickler

**Affiliations:** Department of Medicine, Division of Medical Oncology, Duke University, Durham, NC, United States; Department of Pharmacology and Cancer Biology, Duke University, Durham, NC, United States; Department of Medicine, Division of Medical Oncology, Duke University, Durham, NC, United States; Duke Cancer Institute, Durham, NC, United States; Department of Medicine, Mayo Clinic, Jacksonville, FL, United States

**Keywords:** circulating tumor DNA, molecular residual disease, colorectal cancer, gastrointestinal cancer

## Abstract

Gastrointestinal (GI) cancers represent a highly lethal subset of human malignancies. Colorectal cancer (CRC) remains the most prevalent subtype, representing the second-leading cause of cancer death in the USA, with rising incidence in younger adults. To improve outcomes for patients with GI malignancies, use of assays to monitor clinically undetectable molecular residual disease (MRD) via the analysis of circulating tumor DNA has become commonplace. However, rigorous studies evaluating the utility of MRD assays have lagged behind their widespread clinical adoption in the USA. We provide a narrative review of clinical studies characterizing the prognostic performance of MRD assays for patients with non-metastatic CRC and evaluate the evidence supporting use of MRD testing to de-escalate or intensify treatment. We additionally highlight emerging evidence and ongoing studies in populations of special interest, including patients with microsatellite instability-high disease and patients with a targetable molecular alteration. Finally, we summarize emerging MRD data for pancreatic, gastroesophageal, and hepatobiliary malignancies. This review offers a comprehensive evaluation of current MRD evidence for GI malignancies and outlines future pivotal trials that will ultimately inform guidelines and direct patient management.

## Introduction

Colorectal cancer (CRC) is the second-leading cause of cancer-related death in the USA, with >155,000 new cases and 55,230 deaths in 2026 [1]. Over the past four decades, expanded access to screening colonoscopy and improved oncologic care have decreased CRC incidence and mortality in the USA, particularly in older patients. However, these advancements have been counterbalanced by increased rates of CRC in people under the age of 50 years. CRC is now the leading cause of cancer-related death in Americans under the age of 50 years and remains a significant public health burden [1]. Novel technologies that enhance prevention, detection, and treatment are needed to further reduce CRC incidence and mortality.

Advances in bioinformatics and diagnostics have spurred the development of highly sensitive molecular residual disease (MRD) assays. These tests detect fragments of cancer-derived molecules in patient blood, even when other standard-of-care testing modalities (imaging, endoscopy, etc.) detect no macroscopic disease [2, 3]. In the USA, MRD tests are now routinely used to inform various clinical decision points, including the confirmation of disease clearance after surgery, adjuvant chemotherapy decision-making, treatment-response monitoring, and the early detection of recurrent disease. Despite the absence of National Comprehensive Cancer Network (NCCN) or other established national guidelines supporting the routine use of MRD testing [4], these assays have gained broad acceptance by patients and clinicians, and are now commonly used [5]. The increased use of MRD assays, often before prospective evidence validates clinical utility, highlights a significant unmet clinical need. Clinicians and their patients are seeking technology that reduces diagnostic uncertainty, guides clinical decision-making, and improves cancer outcomes. In a survey of patients with resected colon cancer who received MRD testing, 96% of patients valued the additional information they received [6]. For clinicians, MRD testing can provide invaluable prognostic information and, in select circumstances, influences therapeutic decision-making [7].

The evidence base supporting MRD testing as a diagnostic tool is evolving rapidly, often leaving clinicians and patients uncertain about how to interpret and apply emerging data. This review evaluates, from a primarily US perspective, available MRD testing options, their prognostic and predictive value, and the evidence supporting treatment escalation and de-escalation strategies. It also explores MRD applications across other GI malignancies and considers how second-generation assays may shape future clinical practice.

## Technical overview of testing circulating tumor DNA

DNA is constantly shed into circulation from tumors via a variety of mechanisms, including metastatic invasion of intact cells, apoptotic sloughing, and exaocytosis [8]. Circulating tumor DNA (ctDNA) is an umbrella term that refers to any tumor-derived DNA that can be sampled in patient blood. Previously described components of ctDNA include cell-free DNA (cfDNA), intact circulating tumor cells (CTCs), and red blood cell or platelet bound DNA [9–12]. Tumor-derived DNA can be distinguished from benign sources based on the presence of oncogenic mutations, distinct methylation profiles, and fragment size [13, 14]. Due to the relative enrichment of tumor-to-healthy-cell DNA in patient plasma, nearly all commercially available ctDNA assays target cfDNA after specialized blood collection that prevents contamination of the plasma layer (Fig. 1). Previous analyses have found the half-life of ctDNA to be exceptionally short, ranging from 15 min to 2 h, as it is rapidly cleared by circulating DNases and metabolized by the liver, spleen, and kidneys [15, 16]. This allows ctDNA to be a highly dynamic marker, with levels falling shortly after surgical resection of localized disease or treatment with effective systemic therapy.

**Figure 1:**
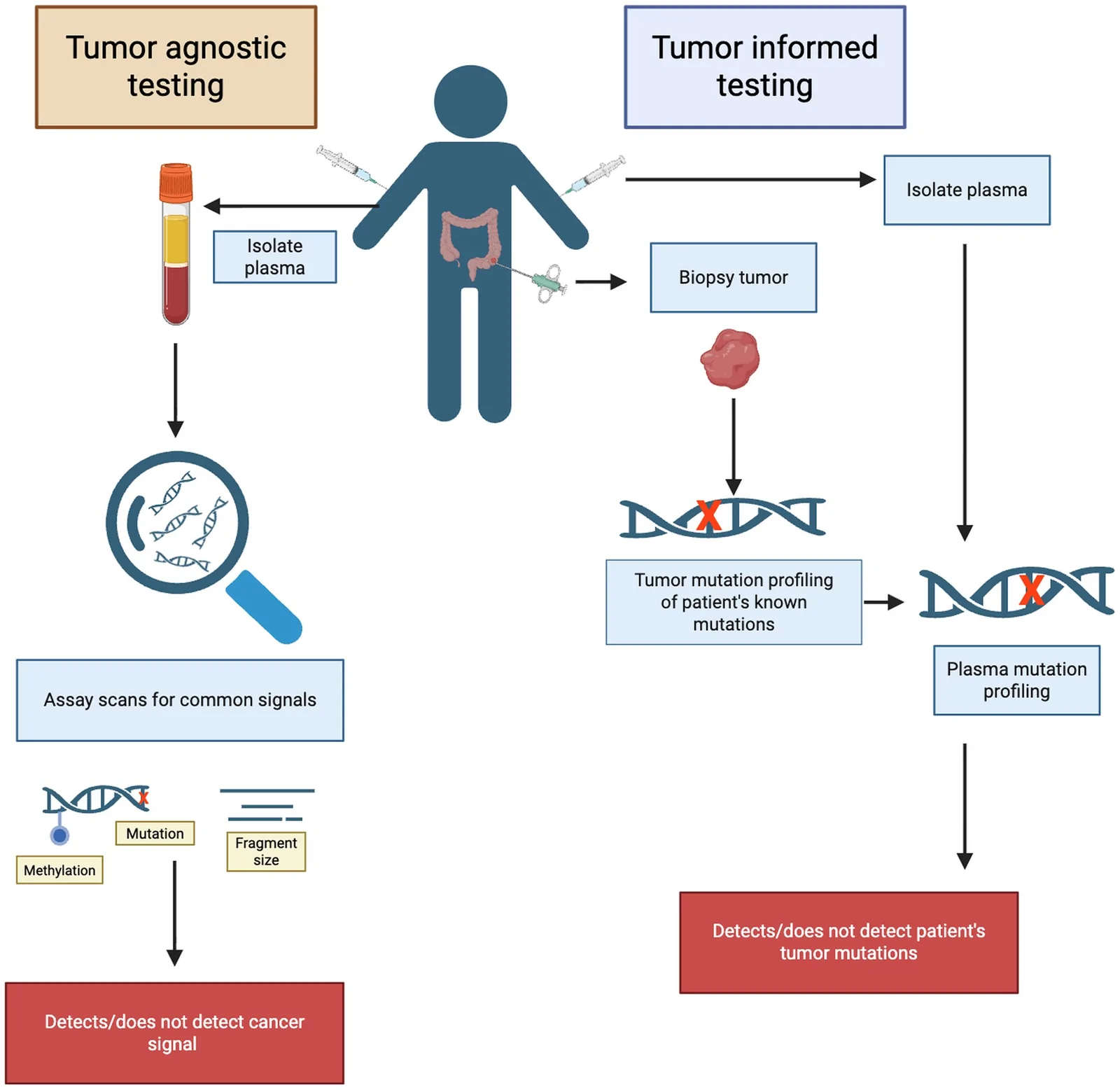
Schema describing the various approaches to assaying circulating tumor DNA in gastrointestinal cancer patients. Approaches can be divided into tumor-agnostic (left) and tumor-informed (right) approaches.

Accurately profiling ctDNA presents a significant technologic challenge, as the proportion of cfDNA that is derived from tumor cells often falls below the limit of detection of conventional Next Generation Sequencing (NGS). The two major approaches to mutation detection involve polymerase chain reaction (PCR) or NGS. Two of the most sensitive forms of PCR-based assays include (i) digital droplet PCR (ddPCR), which involves the microfluidic preparation of individual DNA fragment PCR reactions within water-in-oil droplets, and (ii) beads, emulsification amplification, and magnetics (BEAMing), which involves performing PCR reactions on primer bound beads followed by fluorescent hybridization [17–19]. These PCR-based approaches enable sensitive mutation detection to variant allele frequencies in the 0.1%–0.01% range. However, PCR-based approaches often can only target a limited number of mutations in specific gene regions. The second major platform for mutation detection is NGS [17–19]. Major advances in library preparation that involve molecular barcoding and joint mutation calling from the forward and reverse DNA strands have improved the sensitivity of NGS [20–22]. This form of error correction allows highly sensitive mutation profiling, with limits of detection reported as variant allele frequences of <0.01%. A potential advantage of NGS is that it does not require specific primers to target a small target region and thus can be used to profile a large number of genes or the entire genome [17–19].

Shedding of ctDNA into the bloodstream is particularly low at specific disease sites such as the lungs, peritoneum, and bones, while ctDNA shedding is highest from lymph node and liver metastases [23, 24]. Further, non-malignant processes, such as clonal hematopoiesis of indeterminate potential (CHIP), can shed potentially confounding mutations from white blood cells and other organs at allele frequencies comparable to that expected from tumor-derived DNA, leading to false-positive results [25]. In fact, spurious MRD detection that spontaneously clears has been reported in ≤2% of cases [26].

To further improve performance, many assays use a tumor-informed approach in which tumor tissue is initially sequenced to identify clonal mutations, followed by targeted quantification of the tumor-defined mutations in circulation [27]. Use of a tumor-informed approach presents challenges that must be weighed against potential assay performance advantages. In terms of logistical feasibility, tumor-agnostic tests typically result faster, do not require access to tumor tissue, and can be less expensive [28]. Thus, in a clinical scenario in which a rapid result is needed for a patient without sufficient biopsy or resection material available, a tumor-agnostic test may be the most appropriate. Conversely, in the adjuvant setting in which ample resected tumor tissue is available and immediate test results may not be needed, a tumor-informed test is likely preferred. Tumor-informed assays are generally preferred in the absence of logistical barriers, due to generally favorable test characteristics, particularly when used for the longitudinal monitoring of ctDNA. Persistent detection of ctDNA is a stronger predictor of recurrence than detection at a single postoperative time point [27, 29]. A comprehensive meta-analysis of MRD assays for CRC recurrence found that the sensitivity of serial tumor-informed assays was significantly higher than that of serial tumor-agnostic tests (0.88 vs 0.59, *P *= 0.001) with comparable false-positive rates (0.09 vs 0.12, *P *= 0.419) [30]. However, when used for only a single time point, both the sensitivity and the false-positive rates were comparable between the tumor-informed and tumor-agnostic approaches.

Available assays also vary in the breadth of the tumor genome targeted, ranging from tests capturing only a few frequently mutated hotspots to whole-exome or whole-genome sequencing [31, 32]. Broader whole-genome sequencing allows sampling of the widest number of distinct clonal mutations, including passenger mutations that may be less likely to vary in frequency with anticancer therapy. Conversely, targeted approaches can be less expensive and allow improved coverage of specific mutations of interest.

In summary, the components most critical to interpreting the ctDNA assay used in a given clinical study are as follows:

- the cancer-specific feature targeted (whole genome vs targeted mutations, DNA methylation, or fragment size);
- the use of a tumor-informed approach targeted at features specifically identified by prior tissue-based sequencing or a tumor-agnostic approach that evaluates common mutational or epigenetic changes;
- the timing (MRD window versus more distant surveillance) and longitudinal nature of patient testing.

Importantly, the performance characteristics of any individual assay will be unique to the sequencing technology and testing approach used, which makes extrapolating results from studies using different assays difficult. While rigorous cross-assay comparisons have not been feasible to date, in most settings, the use of longitudinal, tumor-informed assays improves prognostic performance, but increases assay cost, logistical complexity, and processing time.

## MRD as a prognostic marker

In comparison with other clinicopathologic features (T-stage, N-stage, lymphovascular invasion, etc.), persistent MRD-positivity after CRC resection is the strongest and most consistent prognostic biomarker for recurrence [26]. The Alliance N0147 trial, which randomized patients to either adjuvant FOLFOX alone or in combination with cetuximab, collected plasma from 2,260 patients with Stage III colon cancer after surgical resection and assessed MRD status by using a tumor-agnostic test [33, 34]. After a median follow-up of 6.1 years, postoperative MRD-positivity was significantly associated with shorter disease-free survival (DFS, hazard ratio [HR] = 5.03, *P *< 0.0001) and overall survival (OS, HR = 4.45). A separate, single-center study of 103 patients with Stage I–IV CRC who underwent curative-intent surgery found that all 15 evaluable patients who had a positive MRD result after the completion of definitive treatment experienced disease recurrence (HR = 11.28, *P *< 0.0001) [35]. The median time to recurrence when using this tumor-agnostic blood test was 162 days.

In observational studies, tumor-informed MRD assays have also demonstrated strong prognostic performance. The INTERCEPT study performed serial, tumor-informed ctDNA testing every 3 months post curative-intent resection for >1,000 US patients with Stage II–IV CRC [36, 37]. An interim analysis found that patients with at least one positive ctDNA result had significantly shorter DFS than those with negative ctDNA results (HR = 17.67, *P *< 0.0001) and the ctDNA status was a stronger predictor of recurrence risk than the disease stage. Notably, the majority of ctDNA-positive patients in the INTERCEPT study had radiographic evidence of disease within 90 days of first detection, suggesting that reflexive imaging after an MRD-positive result may reduce the lead time to radiographic detection. BESPOKE CRC was a multicenter, observational study that also explored the utility of longitudinal, tumor-informed ctDNA testing in a cohort of >1,000 US patients who previously underwent the resection of Stage II–III CRC [7]. The results again confirmed a strong association between ctDNA-positivity and inferior DFS for patients with both Stage II (HR = 10.4, *P *< 0.0001) and Stage III (HR = 10.1, *P *< 0.0001) diseases. Assay sensitivity varied widely based on the pattern of metastatic spread, with ctDNA-positivity observed in 50% (3/6) of local recurrences, 76% (13/17) of lung recurrences, and 96% (52/54) of liver recurrences. Longitudinal ctDNA-clearance patterns after adjuvant chemotherapy also strongly predicted recurrence risk. Specifically, patients who exhibited sustained ctDNA clearance experienced the lowest risk of recurrence, while patients with transient or no observed clearance experienced the highest recurrence risk. Importantly, these associations between ctDNA-positivity and risk of recurrence are not specific to US cohorts and have also been reported in other international studies, most prominently in Europe and Japan [23, 26, 27, 29].

Despite its strength as a prognostic biomarker, the clinical utility of ctDNA testing remains unclear. Ideally, ctDNA surveillance would increase the detection of low-burden, oligometastatic disease that is amenable to curative-intent local therapy. However, a retrospective analysis of 184 patients treated at a single US center found that only 11 of 20 patients with detectable ctDNA prior to radiographic recurrence were able to undergo metastasectomy and only 3 patients (1.6% of the total population) had durable DFS after resection [38]. Due to concerns regarding clinical utility, additional evidence is needed to determine the role for ctDNA-based MRD testing for routine surveillance and treatment decision-making [4]. Key studies describing the prognostic significance of MRD-positivity are summarized in Table 1.

**Table 1:** Notable studies investigating MRD performance in CRC.

**Study**	Trial name	Study population	ctDNA assay design	Key results
**Observational or post-hoc studies**				
Reinert T, *et al.* 2019	Multicenter study in Denmark	Stage I–III CRC (*n *= 130)	Tumor-informed Longitudinal testing	MRD post operation is associated with increased recurrence risk (HR 7.2) MRD post adjuvant chemotherapy is associated with increased recurrence risk (HR 17.5) Adjuvant chemotherapy can clear MRD
**Parikh AR, *et al.* 2021**	**Single-center US study**	**Stage I**–**IV CRC (*n *= 103)**	**Tumor-agnostic** **Longitudinal testing**	**MRD is associated with recurrence risk (HR 11.28, PPV 100%)**
**Maddalena G, *et al.* 2024**	**INTERCEPT**	**Stage II**–**IV CRC (*n *= 1,140)**	**Tumor-informed** **Longitudinal testing**	**MRD is associated with shorter DFS (HR 17.67)** **MRD-negative patients have a low risk of recurrence (NPV 98.1%)**
Nakamura Y, *et al.* 2024	CIRCULATE-Japan GALAXY	Stage II–III colon or Stage IV CRC (*n *= 2,240)	Tumor-informed Longitudinal testing	MRD is associated with shorter DFS (HR 11.99) and OS (HR 9.68) MRD clearance lengthens DFS
Slater S, *et al.* 2024	UK TRACC Part B	Stage I–III CRC (*n *= 214)	Tumor-agnostic Longitudinal testing	MRD is associated with lower 2-year RFS rate (50.4% vs 91.1%) MRD-negative patients have a low risk of recurrence post definitive treatment (NPV 91.2%)
**Shah PK, *et al.* 2025**	**BESPOKE CRC**	**Stage II**–**III colorectal cancer (*n *= 1,001)**	**Tumor-informed** **Longitudinal testing**	**MRD is associated with shorter DFS (HR 10.4 for Stage II, 10.1 for Stage III)** **MRD clearance lengthens DFS**
**Sinicrope FA, *et al.* 2026**	**Alliance N0147**	**Stage III colon cancer (*n *= 2,260)**	**Tumor-agnostic** **Single time point**	**MRD is associated with shorter DFS (HR 5.03) and OS (HR 4.45)**
**Prospective interventional studies**				
Tie J, *et al.* 2022	DYNAMIC	Stage II colon cancer (*n *= 455)	Tumor-informed Longitudinal testing	ctDNA-guided adjuvant chemotherapy decision-making is non-inferior to adjuvant chemotherapy decision-making based on standard clinicopathologic features
**Morris VK, *et al.* 2024**	**COBRA (NRG-GI005)**	**Low-risk Stage IIA colon cancer (*n *= 635)**	**Tumor-agnostic** **Single time point**	**Adjuvant chemotherapy does not improve MRD clearance**
Bando H, *et al.* 2026	ALTAIR	Stage 0–IV CRC (*n *= 243)	Tumor-informed Longitudinal testing	Six months of FTD/TPI does not significantly improve DFS in MRD-positive patients
Marsoni S, *et al.* 2025	PEGASUS	T4N0 or Stage III colon cancer (*n *= 135)	Tumor-agnostic Limited time points	12% recurrence rate observed in 100 ctDNA-negative patients treated with 6 months of capecitabine
**Pellatt AJ, *et al.* 2025**	**INTERCEPT-TT**	**Stage II**–**IV CRC (*n *= 15)**	**Tumor-informed** **Longitudinal testing**	**Six months of FTD/TPI can clear MRD in 47% and 36% of patients at 3- and 6-month time points, respectively**
Tie J, *et al.* 2025	DYNAMIC-III	Stage III colon cancer (*n *= 968)	Tumor-informed Longitudinal testing	ctDNA-guided adjuvant therapy de-escalation in MRD-negative patients reduced chemotherapy exposure, but did not meet pre-specified non-inferiority compared with standard adjuvant chemotherapy (3-year RFS 85.3% vs 88.1%) ctDNA-guided chemotherapy escalation in MRD-positive patients does not improve RFS relative to standard adjuvant chemotherapy
Folprecht G, *et al.* 2026	CIRCULATE (AIO-KRK-0217)	Stage II pMMR/MSS colon cancer (*n* = 1,396)	Tumor-informed Single time point	Adjuvant chemotherapy does not improve 3-year DFS in MRD-positive patients; however, per-protocol analysis suggests potential benefit (HR 0.31)

Rows in bold text highlight landmark US studies. CRC = colorectal cancer, ctDNA = circulating tumor DNA, DFS = disease-free survival, FTD/TPI = trifluridine/tipiracil, HR = hazard ratio, MRD = molecular residual disease, MSS = microsatellite stable, NPV = negative predictive value, NRG = NRG Oncology, OS = overall survival, pMMR = mismatch repair proficient, PPV = positive predictive value, RFS = recurrence-free survival.

## MRD testing to de-escalate therapy

In observational studies, patients with MRD-negative test results following surgery had a favorable prognosis and did not appear to benefit from adjuvant chemotherapy [7, 26]. Limiting the use of adjuvant chemotherapy in patients with low risk of recurrence is an important goal, as it avoids both acute toxicity and, in the case of oxaliplatin, the significant risk of lifelong peripheral neuropathy [39, 40]. De-escalation strategies also have the potential to reduce healthcare costs; however, these economic benefits are more difficult to quantify and will likely depend on how ctDNA test results are managed [41]. The DYNAMIC trial was one of the first prospective studies to explore MRD testing to guide adjuvant chemotherapy [42]. DYNAMIC was a multicenter, randomized Phase II trial conducted in Australia and New Zealand. The study randomized 455 patients with resected Stage II colon cancer to either ctDNA-guided clinical decision-making, where patients only received adjuvant chemotherapy if ctDNA-positive, or standard management, where adjuvant treatment decisions were based on conventional clinicopathologic criteria. After a median follow-up of nearly 60 months, DYNAMIC found that patients receiving ctDNA-guided management were nearly half as likely to receive adjuvant chemotherapy, but had no difference in 5-year recurrence-free survival (RFS) or OS. In the DYNAMIC-III trial, investigators then evaluated the impact of ctDNA-guided de-escalation in patients with Stage III colon cancer. This study randomized 968 patients to either ctDNA-guided de-escalation or standard management post resection [24]. In the ctDNA-guided arm, patients who were ctDNA-negative received reduced-intensity adjuvant chemotherapy. Notably, this study did not meet its non-inferiority endpoint, as ctDNA-guided de-escalation was associated with slightly lower 3-year RFS (85.3% vs 88.1%). However, a pre-specified subgroup analysis found non-inferiority for ctDNA-guided management in patients with the lowest-risk (T1–3N1) tumors. Similarly, the PEGASUS trial performed tumor-agnostic ctDNA testing on patients with T4N0 or Stage III colon cancer after resection and tailored adjuvant treatment based on ctDNA status. Specifically, patients who were ctDNA-positive received 3 months of adjuvant CAPOX and ctDNA-negative patients received 6 months of single-agent capecitabine [43]. After observing 12 relapses among 100 ctDNA-negative patients, PEGASUS failed to meet its predefined threshold supporting the feasibility of ctDNA-guided adjuvant therapy. The PEGASUS trial’s use of a tumor-agnostic assay raises the question of whether the high recurrence rate in ctDNA-negative patients was driven by de-escalated adjuvant chemotherapy or possibly insufficient assay sensitivity.

DYNAMIC, DYNAMIC-III, and PEGASUS lay the groundwork for ongoing studies that will clarify the role for ctDNA status to de-escalate adjuvant therapy. CIRCULATE-North America (NRG-GI008) is a Phase II/III clinical trial investigating the role of postoperative ctDNA status to guide adjuvant chemotherapy in patients with high-risk Stage II and Stage III colon cancer. In its de-escalation arm, patients with negative MRD test results after resection are randomized to adjuvant oxaliplatin-based chemotherapy versus surveillance alone, with the primary endpoints of time to ctDNA detection and DFS [44]. A similar study, VEGA, is ongoing in Japan and will randomize MRD-negative patients with resected high-risk Stage II or low-risk Stage III colon cancer to surveillance alone versus 3 months of adjuvant CAPOX [45]. Currently, NCCN and ASCO guidelines do not endorse the routine use of ctDNA to inform treatment decision-making. Internationally, guidelines and reimbursement for clinical MRD testing are variable based on the regulatory bodies governing each assay and payor rules (Table 2) [4, 46–49]. The highly anticipated results of CIRCULATE-North America and VEGA will likely clarify the role for MRD testing to guide adjuvant chemotherapy and are likely to impact future practice guidelines.

**Table 2:** Consensus guidelines and regulatory positions on MRD testing in the USA relative to other regions/countries (2026).

Domain	USA	Europe	Japan
Consensus guideline statements on prognostic value	NCCN Colon V2.2026: ctDNA-positivity is prognostic for recurrence	ESMO 2022 (pan-tumor): detection of ctDNA following curative therapy for early-stage cancers has “high evidence of clinical validity” in predicting future relapse	JSMO 2024: MRD testing is strongly recommended for estimating the risk of recurrence
	ASCO 2026 (pan-tumor): ctDNA testing may have clinical validity in predicting the risk of recurrence		
Consensus guideline statements on routine use to guide clinical management	NCCN Colon V2.2026: insufficient evidence to recommend routine use outside of a clinical trial	ESMO 2022 (pan-tumor): there is “no evidence of clinical utility” for routine use in directing treatment	JSMO 2024: MRD is strongly recommended to assess the optimal adjuvant chemotherapy in patients with CRC having received curative resection
	ASCO 2026 (pan-tumor): no recommendation for MRD/surveillance; clinical utility not established		
Governing regulatory framework and reimbursement	Laboratory-developed tests under CLIA or FDA device/companion diagnostic pathway, variable reimbursement	IVDR Class C review required with IVDR Article 5(5) health-institution exemption. There are nation-specific reimbursement patterns	PMDA and MHLW review required. Signatera PMDA-approved for CRC (June 2026); MHLW reimbursement and pricing pending

ASCO 2026 from Lockwood CM, *et al. JCO Oncol Pract* 2026 (PMID 42314080); ESMO 2022 from Pascual J, *et al. Ann Oncol* 2022 (PMID 35809752); JSMO 2022 from Bando H, *et al. Cancer Sci* 2024 (PMID 38263580); NCCN Clinical Practice Guidelines in Oncology, Colon Cancer, Version 2.2026 (7 April 2026). ASCO = American Society of Clinical Oncology, CLIA = Clinical Laboratory Improvement Amendments of 1988, CRC = colorectal cancer, ctDNA = circulating tumor DNA, ESMO = European Society for Medical Oncology, FDA = US Food and Drug Administration, IVDR = EU In Vitro Diagnostic Medical Devices Regulation, JSMO = Japanese Society of Medical Oncology, MHLW = Ministry of Health, Labour and Welfare, MRD = molecular residual disease, NCCN = National Comprehensive Cancer Network, PMDA = Pharmaceuticals and Medical Devices Agency.

## MRD testing to escalate therapy

Several observational studies have suggested that adjuvant chemotherapy is only beneficial in patients with MRD-positive CRC. In the GALAXY study, patients with high-risk Stage II/III colon cancer who were ctDNA-positive following surgery had a statistically significant DFS benefit from adjuvant chemotherapy (adjusted HR = 0.23; *P *< 0.001), but, for those patients who were ctDNA-negative, there was no benefit (adjusted HR = 0.70; *P *= 0.09) [26]. Similarly, in the observational BESPOKE trial, US patients with high-risk Stage II/III colon cancer who were ctDNA-positive following surgery had a statistically significant DFS benefit from adjuvant chemotherapy, but there was no benefit for those patients who were ctDNA-negative [7]. The DYNAMIC trial, which enrolled patients with resected Stage II colon cancer, further supported ctDNA-guided adjuvant chemotherapy [42]. Collectively, these data suggest that ctDNA status could serve as a “tie breaker” to determine whether a patient with Stage II disease should receive adjuvant chemotherapy. However, to date, there is no evidence from a prospective clinical trial that treatment escalation based on ctDNA-positivity improves OS.

Assay characteristics (sensitivity, specificity, positive predictive value, and negative predictive value) significantly impact the results of treatment-escalation trials. In the Phase II/III COBRA trial (NRG-GI005), 635 patients with low-risk Stage IIA colon cancer were randomized to active surveillance (Arm A) or adjuvant FOLFOX/CAPOX if ctDNA-positive (Arm B). This trial utilized a tumor-agnostic assay. The study was closed for futility after the Phase II portion revealed no improvement in the primary endpoint—ctDNA clearance at 6 months in ctDNA-positive patients. Among the 16 ctDNA-positive patients, 3 of 7 patients receiving active surveillance spontaneously cleared their ctDNA, as compared with 1 of 9 patients assigned to adjuvant chemotherapy (*P *= 0.98) [50]. In the COBRA trial, the assay specificity was likely too low for a patient population with low baseline recurrence risk. As a result, the assay likely had too many false positives and an unacceptably high spurious “spontaneous clearance” rate. Future trial designs should either consider multiple assays (“pick the winner design”) or a well-validated tumor-informed assay with high specificity.

The DYNAMIC-III trial further highlights the limitations of MRD testing to guide treatment escalation. In this study, 259 patients with resected Stage III colon cancer were ctDNA-positive immediately following resection and these patients were randomized (1:1) to either ctDNA-guided chemotherapy escalation (*n *= 129) or physician-nominated standard chemotherapy (*n *= 130). Notably, 50% of patients receiving ctDNA-informed escalation received ≥3 months of FOLFOXIRI. Despite the lower-intensity chemotherapy in the standard management group (86% received FOLFOX/CAPOX), the 2-year RFS rate was numerically higher in the standard management group than in the ctDNA-guided chemotherapy-escalation group (61% vs 51%; HR = 1.16) [51]. In a post-hoc analysis, ctDNA clearance—a biomarker associated with clinical benefit—was no more likely in patients receiving FOLFOXIRI than in patients receiving FOLFOX [24]. These results from DYNAMIC-III highlight the limitations of a treatment-escalation strategy. First, effective escalation requires effective treatment. Adjuvant FOLFOXIRI, which is the preferred escalation strategy in DYNAMIC-III, has not demonstrated a survival benefit over CAPOX/FOLFOX. Second, the escalation strategy must overcome the poor prognosis and high recurrence risk for patients with MRD-positive disease [7, 26, 37].

To date, evidence supporting additional systemic treatment following definitive therapy (surgery with or without adjuvant chemotherapy) in patients with MRD-positive disease is limited. ALTAIR (Initial Attack on Latent Metastasis Using TAS-102 for ctDNA-identified Colorectal Cancer Patients After Curative Resection) was a Phase III trial conducted in Japan, in which 243 patients with ctDNA-positive CRC following definitive treatment were randomized to 6 months (six cycles) of trifluridine and tipiracil (FTD/TPI) or placebo. The primary endpoint was DFS. Although the primary analysis demonstrated no DFS improvement with FTD/TPI over placebo [52], a post-hoc central radiographic review found a statistically significant DFS benefit in favor of FTD/TPI [53]. Findings from the ALTAIR trial parallel results from the US-based INTERCEPT-TT trial. In this study, 15 patients with ctDNA-positive CRC following definitive treatment were treated with FTD/TPI and compared with a synthetic control of 30 patients from the INTERCEPT registry [54]. The primary endpoint—6-month ctDNA-clearance rate—was significantly higher in the FTD/TPI group (36%) than in the synthetic control (7%). Median DFS was also longer in patients treated with FTD/TPI (9.4 months) than in the synthetic control (5.75 months) (*P *= 0.03). Findings from INTERCEPT-TT and ALTAIR established the feasibility of treating CRC at molecular rather than radiographic recurrence. There may be other advantages to detecting recurrence early. The FIND trial, which was conducted across multiple centers in China, randomized 584 patients with previously resected Stage I–III CRC to ctDNA-guided surveillance or standard surveillance [55]. Importantly, patients with a positive ctDNA result underwent immediate CT-based imaging followed by bimonthly scans. Use of this ctDNA-triggered surveillance escalation led to a significantly higher proportion of patients receiving curative-intent, metastasis-directed therapy (48.1% vs 23.6%, *P *= 0.008). Longer-term follow-up of this study is pending and whether the delay of radiographic recurrence makes a meaningful impact on survival remains an open question.

Several prospective clinical trials are investigating other treatment-intensification strategies for patients with ctDNA-positivity and no radiographic evidence of disease. CIRCULATE-North America (NRG-GI008) is evaluating adjuvant CAPOX/FOLFOX versus FOLFOXIRI in patients with high-risk Stage II/III colon cancer and ctDNA-positivity following resection [56]. Similarly, the Phase 3 CIRCULATE-PRODIGE 70 trial randomizes patients with ctDNA-positive Stage II colon cancer to either adjuvant mFOLFOX6 or observation [57]. Given prior disappointing results with conventional chemotherapy escalation, the next generation of clinical trials seek to incorporate novel therapeutic strategies, including antibody–drug conjugates, molecularly targeted precision therapies, immunotherapy combinations, and personalized vaccines (Table 3).

**Table 3:** US trials exploring novel or targeted therapies for MRD-positive CRC or GI cancers, per ClinicalTrials.gov, as of 9 September 2026.

Trial identifier	Study phase	Treatment approach	ctDNA assay	Randomization	Disease stage	Planned sample size	Recruitment status	Primary endpoints
**Immunotherapy-based trials**								
NCT06358430	1	TROP2-CAR-NK cells and cetuximab	Not specified	Single-arm, not randomized	Not specified	10	Active, not recruiting	• Safety • ctDNA clearance at 3 months
NCT07071961	2	Lorigerlimab (PD-1 and CTLA-4 inhibitor) and regorafenib	Any CLIA-certified assay for enrollment. Confirmed with tumor-informed assay prior to treatment	Single-arm, not randomized	Resected Stage II–IV CRC	16	Recruiting	• ctDNA clearance at 6 months
NCT07136077	2	Tislelizumab (PD-1 inhibitor) and fruquintinib	Tumor-informed assay	Single-arm, not randomized	Stage II–IV CRC post curative-intent treatment	15	Active, not recruiting	• ctDNA clearance at 6 months
NCT07058012	2	Cemiplimab (PD-1 inhibitor), fianlimab (LAG-3 inhibitor), and REGN7075 (EGFR+CD28 bispecific)	Any CLIA-certified assay for enrollment. Confirmed with tumor-informed assay prior to treatment	Not randomized	Stage II–IV CRC post curative resection and/or ablation	79	Recruiting	• ctDNA clearance at 12 weeks
NCT07227636	2	Botensilimab (CTLA-4 inhibitor) and balstilimab (PD-1 inhibitor)	Not specified	MRD^+^ patients randomized to botensilimab and balstilimab or placebo	Stage III–IV CRC post curative resection and/or ablation	284	Recruiting	• ctDNA clearance at 6 months • 1-year RFS
NCT05482516	2	Atezolizumab (PD-L1 inhibitor) and bevacizumab	Tumor-informed assay	Not randomized	Any gastrointestinal cancer treated with curative intent	20	Recruiting	• ctDNA clearance at 12 weeks • Partial ctDNA response at 12 weeks • ctDNA progression at 12 weeks
**Molecularly targeted trials**								
NCT07023289	2	Telisotuzumab adizutecan (MET-targeted ADC)	Tumor-informed assay	MRD^+^ patients randomized to study drug or standard of care	Any stage of CRC post curative-intent treatment	140	Recruiting	• DFS
NCT03803553	2	Nivolumab, encorafenib/binimetinib/cetuximab, trastuzumab/pertuzumab or standard management based on molecular profile	Tumor-agnostic assay	• Biomarker-negative: MRD^+^ patients randomized to FOLFIRI vs surveillance • Biomarker-positive: all MRD^+^ patients given biomarker-directed therapy	Resected Stage III colon cancer	400	Recruiting	• DFS • ctDNA clearance
NCT06878131	Observational	BRAF + MEK inhibitor, KRAS G12C + EGFR inhibitor, PD-1 +/− CTLA-4 inhibitor, or HER2 inhibitor based on molecular profile	Not specified	Not randomized	Stage II–IV CRC post curative-intent treatment	40	Recruiting	• Safety
NCT06617858	2	AMB-05X (monoclonal CSF1R antibody)	Tumor-informed assay	Not randomized	Stage I–IV CRC post curative-intent treatment	15	Recruiting	• ctDNA clearance at 6 months
**Novel treatment combinations**								
NCT04920032	1b	FTD/TPI and irinotecan	Tumor-informed assay	Not randomized	Stage II–IV CRC post curative-intent treatment	22	Recruiting	• ctDNA-positivity after six cycles
NCT05343013	2	FTD/TPI (completed) FTD/TPI plus celecoxib	Any CLIA-certified assay for enrollment. Confirmed with tumor-informed assay prior to treatment	Not randomized	Stage II–IV CRC post resection	25	Recruiting	• ctDNA clearance after 6 months
**Personalized vaccine**								
NCT04486378	2	RO7198457 (personalized mRNA vaccine)	Not specified	MRD^+^ patients randomized to RO7198457 over 12 months vs observation	Stage II–III CRC post resection	327	Terminated (August 2026)	• DFS

ADC = antibody–drug conjugate, BRAF = B-Raf proto-oncogene, serine/threonine kinase, CAR-NK = chimeric antigen receptor natural killer (cell), CD28 = cluster of differentiation 28, CLIA = Clinical Laboratory Improvement Amendments of 1988, CRC = colorectal cancer, CSF1R = colony-stimulating factor 1 receptor, ctDNA = circulating tumor DNA, CTLA-4 = cytotoxic T-lymphocyte-associated protein 4, DFS = disease-free survival, EGFR = epidermal growth factor receptor, FTD/TPI = trifluridine/tipiracil, GI = gastrointestinal, HER2 = human epidermal growth factor receptor 2, KRAS = Kirsten rat sarcoma viral oncogene homolog, LAG-3 = lymphocyte-activation gene 3, MEK = mitogen-activated protein kinase kinase, MET = mesenchymal–epithelial transition factor, MRD = molecular residual disease, mRNA = messenger ribonucleic acid, NCT = National Clinical Trial (ClinicalTrials.gov identifier), PD-1 = programmed cell death protein 1, PD-L1 = programmed death-ligand 1, TROP2 = trophoblast cell surface antigen 2.

For patients with Stage III colon cancer who are ctDNA-positive following resection, MRD-positivity presents a clinical dilemma. There is currently no evidence that treatment intensification improves survival. Additionally, many patients are either unable or unwilling to enroll in a trial. For these patients, current guidelines offer no consensus approach. A post-hoc analysis from the Phase III CALGB/SWOG 80702 clinical trial suggests that the COX-2 inhibitor/NSAID celecoxib could offer a DFS and OS benefit. In this study, 2,526 patients with resected Stage III colon cancer were randomized to 3 years of celecoxib versus placebo and 3 versus 6 months of adjuvant FOLFOX [58]. In the primary analysis, celecoxib failed to demonstrate a DFS benefit (HR = 0.89; *P *= 0.12). In the post-hoc analysis, 940 evaluable patients had blood and tissue available for tumor-informed MRD testing, of whom 18.4% had ctDNA-positivity immediately following surgery. In this ctDNA-positive patient population, celecoxib was associated with improved 3-year DFS (adjusted HR [aHR], 0.61; 95% CI, 0.42–0.89) and OS (aHR, 0.62; 95% CI, 0.40–0.96) compared with placebo. Notably, ctDNA-negative patients experienced no benefit from celecoxib. However, these results have several caveats. First, this was an unplanned, post-hoc analysis. Second, nearly two-thirds of the enrolled patients were excluded from the analysis, potentially introducing selection bias. Finally, the biological mechanism explaining the survival benefit of COX-2 inhibition in patients with ctDNA-positivity is unknown. Due to these important caveats, prospective validation of these findings is needed.

## MRD testing to assess treatment response in molecularly selected CRC

A key limitation of treatment escalation in patients with MRD-positive CRC is the lack of effective treatment strategies. An important exception is microsatellite instability-high (MSI-H) or mismatch repair deficient (dMMR) CRC, for which immune checkpoint inhibition (ICI) is known to be highly effective [59–61]. For patients with dMMR/MSI-H CRC, MRD testing has emerged as a complementary assay to carcinoma embryonic antigen (CEA), endoscopy, and radiographic surveillance. Importantly, all studies evaluating the utility of MRD testing in MSI-H CRC reported to date have been conducted in patients with clinical or radiographic evidence of advanced disease and MRD results have been evaluated as a marker of molecular response rather than a marker of occult disease. The clinical utility of tumor-informed MRD testing to assess ICI treatment response was initially established by the INSPIRE trial. In this Phase II multi-cohort study, 94 patients with advanced solid tumors were treated with pembrolizumab and followed for ctDNA response. Notably, ctDNA kinetics tracked closely with radiographic response and, after a median follow-up of 25.4 months, patients who cleared their ctDNA had 100% OS [62]. Results from the French SAMCO-PRODIGE 54 trial further validated ctDNA as a predictor of ICI response in patients with MSI-H/dMMR mCRC. In this study, ctDNA change 1 month after the start of ICI was strongly associated with progression-free survival and OS [63]. Recently, a retrospective analysis of US payer claims data noted a significant association between ctDNA clearance and long-term survival in patients with MSI-H CRC treated with first-line ICI [64].

In the neoadjuvant setting, several studies have shown that ctDNA clearance strongly predicts durable treatment response. In a Phase II study, 35 patients with non-metastatic dMMR/MSI-H solid tumors (including CRC) were treated with pembrolizumab [65]. Decline of ctDNA after 3 weeks of treatment was strongly associated with radiographic and pathologic response. Long-term follow-up of patients who experienced clearance of their ctDNA revealed 3-year EFS of 80% (95% CI: 66%–93%) and 3-year OS 94% (95% CI: 86%–100%) [66]. Finally, in the NICHE-2 trial, 115 patients with locally advanced dMMR/MSI-H colon cancer received neoadjuvant ipilumumab and nivolumab, and 102 patients (92%) had ctDNA-positivity prior to treatment. After only 2 weeks, nearly half of patients had ctDNA clearance and, by the time of surgery, >80% of patients had ctDNA clearance; 94% of patients with a pathologic complete response (pCR) experienced ctDNA clearance, whereas persistent ctDNA-positivity was associated with nonresponse [59]. In the USA, where the NICHE-2 neoadjuvant treatment strategy is increasingly utilized, clinicians are seeking diagnostic tools to assess treatment response. Given the strong association between ctDNA clearance and durable ICI response in patients with MSI-H CRC, MRD testing is increasingly utilized to guide management decisions, particularly as imaging and endoscopic surveillance are notoriously unreliable. An important future research direction will be to evaluate whether ctDNA status after neoadjuvant immunotherapy and longitudinal MRD monitoring can be used to guide surgical management, with ctDNA-negative patients potentially avoiding surgery entirely. Given the large number of immunotherapy trials currently open and in development for ctDNA-positive and radiographically negative CRC (Table 3), MRD testing represents an important diagnostic tool that may one day support Food and Drug Administration registration.

Beyond MSI-H/dMMR colon cancer, other precision medicine strategies to treat ctDNA-positive colon cancer are in development. One such observational study, PRECISION-MRD, is enrolling patients with ctDNA-positivity and no radiographic evidence of disease after locoregional treatment. Patients are treated with biomarker-directed therapy, as determined by the treating physician, targeting *BRAF*^V600E^, *KRAS*^G12C^, and *HER2* amplification (NCT06878131). Given the limited treatment options and poor prognosis for patients with ctDNA-positivity, these targeted approaches offer innovative strategies to address an unmet need.

## MRD testing in other GI cancers

While progress in the clinical implementation of ctDNA testing has been greatest in CRC, efforts to evaluate the utility of ctDNA monitoring in additional cancer types is underway. Essential elements of a successful MRD test in a new cancer type include (i) an assay with the sensitivity to detect ctDNA in a patient with minimal disease burden and (ii) a clinical scenario for which ctDNA detection could meaningfully inform management. Both elements remain in question for pancreatic cancer. The low mutation burden and low amount of ctDNA shedding exhibited by pancreatic tumors present a significant technologic challenge for current ctDNA assays [67, 68]. This was exemplified by a study that performed serial tumor-informed ctDNA monitoring, including a preoperative time point, in 35 patients who underwent pancreatic cancer resection [69]. This study found that only eight patients (25% of the total cohort) had measurable ctDNA at the preoperative time point despite all patients having residual tumor after pathologic review. The emergence of detectable ctDNA after resection was a significant negative prognostic marker (median OS for ctDNA-positive, 110 days versus ctDNA-negative, 381 days); however, only 50% of patients who recurred within the study period had detectable ctDNA at the time of recurrence. One promising strategy to improve the sensitivity of ctDNA testing for pancreas cancer is focusing assays on recovering rare DNA fragments with KRAS and other ubiquitous hotspots [32, 70, 71]. Even if adequate assay sensitivity is achieved, it is unclear whether MRD detection could meaningfully inform the postoperative management of pancreas cancer, as the majority of patients who undergo curative-intent resection ultimately recur [72]. The DYNAMIC-Pancreas trial randomized 102 patients with pancreas cancer to adjuvant gemcitabine doublet versus FOLFIRINOX, with MRD-positive patients receiving 6 months of therapy and MRD-negative patients receiving 3–4 months of therapy. There were no significant differences in RFS observed between the trial arms. In the ctDNA-negative group, there was a nonsignificant trend toward improved RFS in patients who received 6 months of therapy (HR 0.64, *P *= 0.159) and FOLFIRINOX (HR = 0.63, *P *= 0.09), suggesting that therapy de-escalation in ctDNA-negative patients may not be appropriate for pancreas cancer [73]. This paradigm is likely to change with the recent approval of daraxonrasib [74], which is actively being investigated in the adjuvant setting in the RASolute 304 trial (NCT07252232).

Gastroesophageal cancer is another malignancy for which ctDNA detection may have clinical utility. A retrospective analysis of 36 patients with resected Stage I–III gastroesophageal cancer found that 81% of evaluable patients had ctDNA detected via a tumor-informed ctDNA assay prior to developing clinical evidence of recurrence [75]. Moreover, this observation that ctDNA-positivity can consistently precede clinical or radiographic evidence of recurrence has been replicated in additional retrospective analyses [76, 77]. Further motivating the investigation of MRD testing, there are multiple clinical scenarios for which MRD detection could be relevant for gastroesophageal cancers, including monitoring for recurrence in patients undergoing “watch-and-wait” organ-preservation strategies and informing patient selection for the adjuvant use of molecularly targeted therapies [78, 79].

Hepatobiliary cancers may also be well suited for ctDNA monitoring, as liver metastases are known to generate high ctDNA shedding [7]. Indeed, the STAMP trial, which compared adjuvant capecitabine with gemcitabine plus cisplatin in 101 patients with resected biliary tract cancer, performed a retrospective evaluation of ctDNA across both study arms. Notably, ctDNA detection was strongly associated with shorter DFS, particularly in patients with sustained positivity throughout adjuvant treatment (HR = 6.7, *P *< 0.001) [80]. Similarly, in a retrospective analysis of 171 patients with resected biliary tract cancer, postoperative ctDNA status was the most significant predictor of DFS (HR = 10.91, *P *< 0.001), even after adjustment for other clinicopathologic variables [81]. Similar trends were observed in patients undergoing curative-intent treatment for localized hepatocellular carcinoma [82–84]. Similarly to gastroesophageal cancer, multiple effective immunotherapy and targeted therapy options are currently approved for patients with advanced-stage hepatobiliary cancer. For these patients, ctDNA status has the potential to support adjuvant trials, particularly for patients with the highest risk for disease recurrence.

In summary, ctDNA monitoring has demonstrated initial promise in pancreatic, gastroesophageal, and hepatobiliary malignancies. However, current evidence is limited to small, retrospective single-center studies. To demonstrate clinical utility, prospective, multicenter studies that evaluate the use of MRD detection to inform treatment decision-making are urgently needed.

## Conclusions

MRD assays have the potential to revolutionize cancer surveillance and treatment selection, but their rapid clinical adoption in the USA precedes consensus national guidelines and clinical trials confirming clinical utility. Further complicating broad adoption, there are limited trials comparing the performance characteristics (sensitivity, specificity, etc.) of commercially available assays and the constant evolution of these assays make any direct comparisons rapidly obsolete. MRD assays are a powerful prognostic tool that has already demonstrated clinical utility for some patients with Stage II colon cancer. However, for patients with resected Stage III colon cancer, there is no evidence that MRD testing can be used to escalate or de-escalate adjuvant therapy. Additionally, there is no evidence that systemic therapy can improve survival for patients who have completed surgery and adjuvant chemotherapy and remain MRD-positive. Given the limited systemic therapy options for patients with persistent MRD-positivity, intensified imaging surveillance (CT, PET, or MRI) may enable potentially curative, locally directed treatment (surgery, radiation, or ablation); however, no prospective trial to date has found an improvement in patient survival associated with use of MRD testing and the optimal surveillance modality, interval, and duration have yet to be defined for MRD-positive patients. There are other scenarios, such as MSI-H/dMMR CRC treated with ICI, in which MRD testing is a helpful complement to routine clinical care. Additionally, MRD testing may have value in pancreatic, gastroesophageal, and hepatobiliary malignancies, though prospective clinical validation is needed. In all cases, the benefits of MRD testing must be weighed against the potential harms and associated costs. Nonetheless, future breakthroughs in cancer treatment will increasingly rely on MRD testing—both to confirm disease response and to identify patients for adjuvant therapy. As MRD assays and cancer therapies continue to improve, the optimal settings for MRD testing and its associated clinical impact will become increasingly clear.

## References

[1] Siegel RL , Wagle NS, Star J et al Colorectal cancer statistics, 2026. CA A Cancer J Clinicians 2026;76:e70067. 10.3322/caac.70067 PMC12951547 41769777

[2] Tie J , Wang Y, Tomasetti C et al Circulating tumor DNA analysis detects minimal residual disease and predicts recurrence in patients with stage II colon cancer. Sci Transl Med 2016;8:346ra92. 10.1126/scitranslmed.aaf6219 PMC5346159 27384348

[3] Garcia-Murillas I , Schiavon G, Weigelt B et al Mutation tracking in circulating tumor DNA predicts relapse in early breast cancer. Sci Transl Med 2015;7:302ra133. 10.1126/scitranslmed.aab0021 26311728

[4] National Comprehensive Cancer Network. NCCN Clinical Practice Guidelines in Oncology: Colon Cancer. [cited 2026 May 17]. https://www.nccn.org/guidelines/guidelines-detail? id=1428 (17 May 2026, date accessed).

[5] Pedersen KS , Bucheit LA, Tan BR et al Clinician utilization of a plasma-only, multiomic minimal residual disease (MRD) assay in 2,000 consecutive patients with colorectal cancer (CRC). JCO 2022;40:e15586. 10.1200/JCO.2022.40.16_suppl.e15586

[6] Kasi PM , Aushev VN, Ensor J et al Circulating tumor DNA (ctDNA) for informing adjuvant chemotherapy (ACT) in stage II/III colorectal cancer (CRC): interim analysis of BESPOKE CRC study. JCO 2024;42:9. 10.1200/JCO.2024.42.3_suppl.9

[7] Shah PK , Aushev VN, Ensor J et al Circulating tumor DNA for detection of molecular residual disease (MRD) in patients (pts) with stage II/III colorectal cancer (CRC): final analysis of the BESPOKE CRC sub-cohort. JCO 2025;43:15. 10.1200/JCO.2025.43.4_suppl.15

[8] Diaz LA , Bardelli A. Liquid biopsies: genotyping circulating tumor DNA. J Clin Oncol 2014;32:579–86. 10.1200/JCO.2012.45.2011 PMC4820760 24449238

[9] Lowes LE , Bratman SV, Dittamore R et al Circulating Tumor Cells (CTC) and Cell-Free DNA (cfDNA) Workshop 2016: scientific opportunities and logistics for cancer clinical trial incorporation. Int J Mol Sci 2016;17:1505. 10.3390/ijms17091505 PMC5037782 27618023

[10] Liang N , Jiao Z, Zhang C et al Mature red blood cells contain long DNA fragments and could acquire DNA from lung cancer tissue. Adv Sci (Weinh) 2023;10:e2206361. 10.1002/advs.202206361 PMC9982546 36599687

[11] Murphy L , Inchauspé J, Valenzano G et al Platelets sequester extracellular DNA, capturing tumor-derived and free fetal DNA. Science 2025;389:eadp3971. 10.1126/science.adp3971 PMC7618233 40811534

[12] Leon SA , Shapiro B, Sklaroff DM et al Free DNA in the serum of cancer patients and the effect of therapy. Cancer Res 1977;37:646–50. 837366

[13] Mouliere F , Chandrananda D, Piskorz AM et al Enhanced detection of circulating tumor DNA by fragment size analysis. Sci Transl Med 2018;10:eaat4921. 10.1126/scitranslmed.aat4921 PMC6483061 30404863

[14] Liu MC , Oxnard GR, Klein EA et al; CCGA Consortium. Sensitive and specific multi-cancer detection and localization using methylation signatures in cell-free DNA. Ann Oncol 2020;31:745–59. 10.1016/j.annonc.2020.02.011 PMC8274402 33506766

[15] Diehl F , Schmidt K, Choti MA et al Circulating mutant DNA to assess tumor dynamics. Nat Med 2008;14:985–90. 10.1038/nm.1789 PMC2820391 18670422

[16] Radif D , Lindskrog SV, Nordentoft I et al The impact of kidney, liver, and immune function on circulating tumor DNA detection in muscle-invasive bladder cancer. Eur Urol Oncol 2025;8:1598–606. 10.1016/j.euo.2025.09.012 41073166

[17] de Abreu AR , Wyninckx A, Vandamme T et al Circulating Tumor DNA detection in cancer: a comprehensive overview of current detection methods and prospects. The Oncologist 2025;30:oyaf204. 10.1093/oncolo/oyaf204 PMC12450317 40680231

[18] Chen K , Shields MD, Chauhan PS et al Commercial ctDNA assays for minimal residual disease detection of solid tumors. Mol Diagn Ther 2021;25:757–74. 10.1007/s40291-021-00559-x PMC9016631 34725800

[19] Semenkovich NP , Szymanski JJ, Earland N et al Genomic approaches to cancer and minimal residual disease detection using circulating tumor DNA. J Immunother Cancer. 2023; 11: e006284. 10.1136/jitc-2022-006284 PMC10314661 37349125

[20] Kennedy SR , Schmitt MW, Fox EJ et al Detecting ultralow-frequency mutations by Duplex Sequencing. Nat Protoc 2014;9:2586–606. 10.1038/nprot.2014.170 PMC4271547 25299156

[21] Jee J , Rasouly A, Shamovsky I et al Rates and mechanisms of bacterial mutagenesis from maximum-depth sequencing. Nature 2016;534:693–6. 10.1038/nature18313 PMC4940094 27338792

[22] Cohen JD , Douville C, Dudley JC et al Detection of low-frequency DNA variants by targeted sequencing of the Watson and Crick strands. Nat Biotechnol 2021;39:1220–7. 10.1038/s41587-021-00900-z PMC8627329 33941929

[23] Kataoka K , Mori K, Nakamura Y et al Survival benefit of adjuvant chemotherapy based on molecular residual disease detection in resected colorectal liver metastases: subgroup analysis from CIRCULATE-Japan GALAXY. Ann Oncol 2024;35:1015–25. 10.1016/j.annonc.2024.08.2240 39293512

[24] Tie J , Wang Y, Loree JM et al ctDNA-guided adjuvant chemotherapy escalation in stage III colon cancer: Primary analysis of the ctDNA-positive cohort from the randomized AGITG dynamic-III trial (intergroup study of AGITG and CCTG). JCO 2025;43:3503– 10.1200/JCO.2025.43.16_suppl.3503

[25] Hu Y , Ulrich BC, Supplee J et al False-positive plasma genotyping due to clonal hematopoiesis. Clin Cancer Res 2018;24:4437–43. 10.1158/1078-0432.CCR-18-0143 29567812

[26] Nakamura Y , Watanabe J, Akazawa N et al ctDNA-based molecular residual disease and survival in resectable colorectal cancer. Nat Med 2024;30:3272–83. 10.1038/s41591-024-03254-6 PMC11564113 39284954

[27] Reinert T , Henriksen TV, Christensen E et al Analysis of plasma cell-free DNA by ultradeep sequencing in patients with stages I to III colorectal cancer. JAMA Oncol 2019;5:1124–31. 10.1001/jamaoncol.2019.0528 PMC6512280 31070691

[28] Dong Q , Chen C, Hu Y et al Clinical application of molecular residual disease detection by circulation tumor DNA in solid cancers and a comparison of technologies: review article. Cancer Biol Ther 2023;24:2274123. 10.1080/15384047.2023.2274123 PMC10653633 37955635

[29] Henriksen TV , Tarazona N, Frydendahl A et al Circulating tumor DNA in stage III colorectal cancer, beyond minimal residual disease detection, toward assessment of adjuvant therapy efficacy and clinical behavior of recurrences. Clin Cancer Res 2022;28:507–17. 10.1158/1078-0432.CCR-21-2404 PMC9401484 34625408

[30] Camblor DG , Martínez-Castedo B, Martín-Arana J et al Clinical performance of tumor-informed versus tumor-agnostic ctDNA assays for colorectal cancer recurrence: a systematic review and diagnostic accuracy meta-analysis. Cancer Treat Rev 2026;142:103066. 10.1016/j.ctrv.2025.103066 41401645

[31] Zviran A , Schulman RC, Shah M et al Genome-wide cell-free DNA mutational integration enables ultra-sensitive cancer monitoring. Nat Med 2020;26:1114–24. 10.1038/s41591-020-0915-3 PMC8108131 32483360

[32] Ramaker RC , Zhang Y, Strickler JH et al Profiling KRAS mutations in whole blood by error-corrected maximum depth sequencing. Npj Precis Onc 2026;10:195. 10.1038/s41698-026-01399-w PMC13219511 41933043

[33] Sinicrope FA , Segovia DI, Hardin A et al Tissue-free circulating tumor DNA assay and patient outcome in a phase III trial of FOLFOX-based adjuvant chemotherapy (Alliance N0147). JCO 2025;43:3504. 10.1200/JCO.2025.43.16_suppl.3504 PMC12866954 41616224

[34] Sinicrope FA , Segovia D, Sharma N et al Tissue-free circulating tumor DNA assay and patient outcome in a phase III trial of FOLFOX-based adjuvant chemotherapy (Alliance N0147). J Clin Oncol 2026;44:1401–15. 10.1200/JCO-25-02086 PMC12866954 41616224

[35] Parikh AR , Van Seventer EE, Siravegna G et al Minimal residual disease detection using a plasma-only circulating tumor DNA assay in patients with colorectal cancer. Clin Cancer Res 2021;27:5586–94. 10.1158/1078-0432.CCR-21-0410 PMC8530842 33926918

[36] Dasari A , Bent AH, Alfaro-Munoz K et al; MD Anderson INTERCEPT. Association of positive ctDNA-based minimal residual disease assays during surveillance and undiagnosed concomitant radiographic recurrences in colorectal cancer (CRC): results from the MD Anderson INTERCEPT program. JCO 2023;41:3522. 10.1200/JCO.2023.41.16_suppl.3522

[37] Maddalena G , Pellatt AJ, Eluri M et al INTERCEPT Program of circulating tumor DNA (ctDNA) testing for minimal residual disease (MRD) in colorectal cancer (CRC): results from a prospective clinical cohort. JCO 2024;42:27. 10.1200/JCO.2024.42.3_suppl.27

[38] Ji J , Wang C, Goel A et al Circulating tumor DNA testing in curatively resected colorectal cancer and salvage resection. JAMA Netw Open 2024;7:e2452661. 10.1001/jamanetworkopen.2024.52661 PMC11681374 39729315

[39] Ferrero J-M , Bouriga R, Gal J et al De-escalating anticancer treatment: watch your step. Cancers (Basel) 2025;17:2474. 10.3390/cancers17152474 PMC12346573 40805173

[40] Kaiser K , Lyleroehr M, Shaunfield S et al Neuropathy experienced by colorectal cancer patients receiving oxaliplatin: a qualitative study to validate the Functional Assessment of Cancer Therapy/Gynecologic Oncology Group-Neurotoxicity scale. World J Gastrointest Oncol 2020;12:205–18. 10.4251/wjgo.v12.i2.205 PMC7031152 32104551

[41] Wang H , Kramer A, Greuter MJE et al CtDNA-guided de-escalation of adjuvant chemotherapy in stage III colon cancer: early model-based evaluation of cost-effectiveness. Ther Adv Med Oncol 2025;17:17588359251384238. 10.1177/17588359251384238 PMC12592672 41209620

[42] Tie J , Cohen JD, Lahouel K et al; DYNAMIC Investigators. Circulating tumor DNA analysis guiding adjuvant therapy in stage II colon cancer. N Engl J Med 2022;386:2261–72. 10.1056/NEJMoa2200075 PMC9701133 35657320

[43] Marsoni S. , Prisciandaro M., Tarazona Llavero N. et al 723O post-surgical liquid biopsy-guided treatment of stage III and high-risk stage II colon cancer patients: final results of the PEGASUS trial. Ann Oncol. 2025;36:S474. 10.1016/j.annonc.2025.08.1298

[44] Lieu CH , Yu G, Kopetz S et al NRG-GI008: colon adjuvant chemotherapy based on evaluation of residual disease (CIRCULATE-North America). J Clin Oncol 2025;43:TPS310. 10.1200/JCO.2025.43.4_suppl.TPS310

[45] Taniguchi H , Nakamura Y, Kotani D et al CIRCULATE-Japan: Circulating tumor DNA-guided adaptive platform trials to refine adjuvant therapy for colorectal cancer. Cancer Sci 2021;112:2915–20. 10.1111/cas.14926 PMC8253296 33931919

[46] Lockwood CM , Messersmith HJ, Kim AS et al Circulating tumor DNA testing in solid tumors and lymphoma: ASCO Guideline. JCO Oncol Pract 2026;22:1468–89. 10.1200/OP-26-00311 42314080

[47] Pascual J , Attard G, Bidard F-C et al ESMO recommendations on the use of circulating tumour DNA assays for patients with cancer: a report from the ESMO Precision Medicine Working Group. Ann Oncol 2022;33:750–68. 10.1016/j.annonc.2022.05.520 35809752

[48] Bando H , Yamaguchi K, Mitani S et al Japanese society of medical oncology clinical guidelines: molecular testing for colorectal cancer treatment, 5th edition. Cancer Sci 2024;115:1014–21. 10.1111/cas.16039 PMC10920993 38263580

[49] Wang F , Chen G, Zhang Z et al The Chinese Society of Clinical Oncology (CSCO): Clinical guidelines for the diagnosis and treatment of colorectal cancer, 2024 update. Cancer Commun (Lond) 2025;45:332–79. 10.1002/cac2.12639 PMC11947620 39739441

[50] Morris VK , Yothers G, Kopetz S et al Phase II results of circulating tumor DNA as a predictive biomarker in adjuvant chemotherapy in patients with stage II colon cancer: NRG-GI005 (COBRA) phase II/III study. JCO 2024;42:5. 10.1200/JCO.2024.42.3_suppl.5

[51] Tie J , Wang Y, Loree JM et al; AGITG DYNAMIC-III Study Group (Intergroup Study of the Australasian Gastro-Intestinal Trials Group and Canadian Cancer Trials Group). Circulating tumor DNA-guided adjuvant therapy in locally advanced colon cancer: the randomized phase 2/3 DYNAMIC-III trial. Nat Med 2025;31:4291–300. 10.1038/s41591-025-04030-w 41115959

[52] Bando H , Watanabe J, Kotaka M et al A randomized, double-blind, phase III study comparing trifluridine/tipiracil (FTD/TPI) versus placebo in patients with molecular residual disease following curative resection of colorectal cancer (CRC): The ALTAIR study. J Clin Oncol 2025;43:LBA22. 10.1200/JCO.2025.43.4_suppl.LBA22

[53] Watanabe J , Bando H, Kotaka M et al Post-hoc central radiological review of the ALTAIR study in patients with molecular residual disease (MRD) following curative resection of colorectal cancer (CRC). J Clin Oncol 2026;44:138. 10.1200/JCO.2026.44.2_suppl.138

[54] Pellatt AJ , Bent A, Hornstein N et al Phase II trial of TAS-102 in colorectal cancer patients with circulating tumor DNA-defined minimal residual disease after adjuvant therapy: INTERCEPT-TT. JCO Precis Oncol 2025;9:e2500142. 10.1200/PO-25-00142 40669021

[55] Mo S , Zhou C, Ma M et al Dynamic circulating tumor DNA methylation monitoring guiding postoperative surveillance in nonmetastatic colorectal cancer: a prospective, randomized, phase III FIND trial. J Clin Oncol 2026;JCO2503009. 10.1200/JCO-25-03009 42525894

[56] Sahin IH , Lin Y, Yothers G et al Minimal residual disease-directed adjuvant therapy for patients with early-stage colon cancer: CIRCULATE-US. Oncology (Williston Park) 2022;36:604–8. 10.46883/2022.25920976 36260786

[57] Taïeb J , Benhaim L, Laurent Puig P et al Decision for adjuvant treatment in stage II colon cancer based on circulating tumor DNA: The CIRCULATE-PRODIGE 70 trial. Digest Liver Dis 2020;52:730–3. 10.1016/j.dld.2020.04.010 32482534

[58] Zhang GQ , Meyerhardt JA, Shi Q et al Predictive role of circulating tumor DNA in stage III colon cancer treated with celecoxib: a post hoc analysis of the CALGB (Alliance)/SWOG 80702 phase 3 randomized clinical trial. JAMA Oncol 2026;12:149–58. 10.1001/jamaoncol.2025.5144 PMC12679421 41343176

[59] Chalabi M , Verschoor YL, Tan PB et al Neoadjuvant immunotherapy in locally advanced mismatch repair–deficient colon cancer. N Engl J Med 2024;390:1949–58. 10.1056/NEJMoa2400634 38838311

[60] André T , Shiu K-K, Kim TW et al; KEYNOTE-177 Investigators. Pembrolizumab in microsatellite-instability–high advanced colorectal cancer. N Engl J Med 2020;383:2207–18. 10.1056/NEJMoa2017699 33264544

[61] André T , Elez E, Lenz H-J et al Nivolumab plus ipilimumab versus nivolumab in microsatellite instability-high metastatic colorectal cancer (CheckMate 8HW): a randomised, open-label, phase 3 trial. Lancet 2025;405:383–95. 10.1016/S0140-6736(24)02848-4 39874977

[62] Bratman SV , Yang SYC, Iafolla MAJ et al Personalized circulating tumor DNA analysis as a predictive biomarker in solid tumor patients treated with pembrolizumab. Nat Cancer 2020;1:873–81. 10.1038/s43018-020-0096-5 35121950

[63] Taïeb J , Sullo FG, Lecanu A et al Early ctDNA and survival in metastatic colorectal cancer treated with immune checkpoint inhibitors: a secondary analysis of the SAMCO-PRODIGE 54 randomized clinical trial. JAMA Oncol 2025;11:874–82. 10.1001/jamaoncol.2025.1646 PMC12177728 40531517

[64] Robinson HR , Hornstein NJ, Boland PM et al Real-world outcomes and circulating tumor DNA (ctDNA) dynamics in patients (pts) with microsatellite instability–high (MSI-H) metastatic colorectal cancer (CRC) treated with immune checkpoint inhibitors (ICI). J Clin Oncol 2026;44:3516– 10.1200/JCO.2026.44.16_suppl.3516

[65] Ludford K , Ho WJ, Thomas JV et al Neoadjuvant pembrolizumab in localized microsatellite instability high/deficient mismatch repair solid tumors. J Clin Oncol 2023;41:2181–90. 10.1200/JCO.22.01351 PMC10489404 36623241

[66] LaPelusa M , Qiao W, Iorgulescu B et al Long-term efficacy of pembrolizumab and the clinical utility of ctDNA in locally advanced dMMR/MSI-H solid tumors. Nat Commun 2025;16:4514. 10.1038/s41467-025-59615-3 PMC12081696 40374594

[67] Grunvald MW , Jacobson RA, Kuzel TM et al Current status of circulating tumor DNA liquid biopsy in pancreatic cancer. Int J Mol Sci 2020;21:7651. 10.3390/ijms21207651 PMC7589736 33081107

[68] Gao Q , Zeng Q, Wang Z et al Circulating cell-free DNA for cancer early detection. Innovation (Camb) 2022;3:100259. 10.1016/j.xinn.2022.100259 PMC9133648 35647572

[69] Eckhoff AM , Kanu E, Fletcher A et al Initial report: personalized circulating tumor DNA and survival in patients with resectable pancreatic cancer. Ann Surg Oncol 2024;31:1444–6. 10.1245/s10434-023-14751-2 38170407

[70] Cox M , Wells A, Vitello D et al Ultra-sensitive detection of mutant KRAS in circulating tumor DNA predicts survival in resectable pancreatic adenocarcinoma. Front Oncol 2025;15:1657746. 10.3389/fonc.2025.1657746 PMC13180577 42158852

[71] Bernard V , Kim DU, San Lucas FA et al Circulating nucleic acids are associated with outcomes of patients with pancreatic cancer. Gastroenterology 2019;156:108–18.e4. 10.1053/j.gastro.2018.09.022 PMC6434712 30240661

[72] Agritelley ES , Ramaker RC, Bao M et al Preparing for the inevitable: early comprehensive genomic profiling for patients with localized pancreatic ductal adenocarcinoma. JCO Oncol Pract 2026;22:585–93. 10.1200/OP-25-00548 PMC12626397 41237365

[73] Lee B , Tie J, Wang Y et al The potential role of serial circulating tumor DNA (ctDNA) testing after upfront surgery to guide adjuvant chemotherapy for early stage pancreatic cancer: The AGITG DYNAMIC-Pancreas trial. JCO 2024;42:107– 10.1200/JCO.2024.42.16_suppl.107

[74] Wolpin BM , Park W, Garrido-Laguna I et al; RMC-6236-001 Investigators Daraxonrasib in previously treated advanced *RAS* -mutated pancreatic cancer. N Engl J Med 2026;394:1790–802. 10.1056/NEJMoa2505783 42090791

[75] Mehta R , Rivero-Hinojosa S, Dayyani F et al Circulating tumor DNA informs clinical practice in patients with recurrent/metastatic gastroesophageal cancers. Cancer. 2026; 132: e70242. 10.1002/cncr.70242 PMC12914159 41485117

[76] Yue P , Bie F, Zhu J et al Minimal residual disease profiling predicts pathological complete response in esophageal squamous cell carcinoma. Mol Cancer 2024;23:96. 10.1186/s12943-024-02006-x PMC11084057 38730415

[77] Hu Q , Kimura Y, Ikeda S et al Circulating tumor DNA monitoring detects minimal residual disease and predicts outcomes in patients with esophageal adenocarcinoma or squamous cell carcinoma after esophagectomy. BJC Rep 2025;3:52. 10.1038/s44276-025-00158-x PMC12290085 40707646

[78] Liu W , Zhang B, Wang C et al Organ preservation in esophageal cancer: current strategies, challenges, and future directions. Cancers (Basel) 2025;17:3559. 10.3390/cancers17213559 PMC12606734 41228350

[79] Li W , Wei J, Cheng M et al Unveiling promising targets in gastric cancer therapy: a comprehensive review. Mol Ther Oncol 2024;32:200857. 10.1016/j.omton.2024.200857 PMC11396074 39280587

[80] Yoo C , Jeong H, Jeong JH et al Circulating tumor DNA status and dynamics predict recurrence in patients with resected extrahepatic cholangiocarcinoma. J Hepatol 2025;82:861–70. 10.1016/j.jhep.2024.10.043 39532185

[81] Abdelrahim M , Esmail A, Tin A et al Real-world analysis of ctDNA and other biomarkers in patients with curatively resected stage I-III biliary tract cancer. JCO 2025;43:4130– 10.1200/JCO.2025.43.16_suppl.4130 PMC13459594 42583118

[82] Hu J , Tang H, Jia C-C et al Personalized MRD assessment in perisurgical ctDNA for prognostic prediction in hepatocellular carcinoma. Clin Cancer Res 2025;31:1047–56. 10.1158/1078-0432.CCR-24-1897 39526910

[83] Buonopane IR , Saldanha E, de Menezes JSA et al Circulating tumor DNA as a minimal residual disease assessment and recurrence risk in hepatocellular carcinoma: A systematic review and meta-analysis. J Clin Oncol 2025;43:e15055. 10.1200/JCO.2025.43.16_suppl.e15055 PMC12905406 41402557

[84] Xu Y , Cai J, Zhong K et al Plasma-only circulating tumor DNA analysis detects minimal residual disease and predicts early relapse in hepatocellular carcinoma patients undergoing curative resection. Front Oncol 2023;13:1119744. 10.3389/fonc.2023.1119744 PMC10028131 36959801

